# Notch Signaling Mediates TNF-****α****-Induced IL-6 Production in Cultured Fibroblast-Like Synoviocytes from Rheumatoid Arthritis

**DOI:** 10.1155/2012/350209

**Published:** 2011-12-08

**Authors:** Zhijun Jiao, Wenhong Wang, Jie Ma, Shengjun Wang, Zhaoliang Su, Huaxi Xu

**Affiliations:** ^1^Key Laboratory of Medical Immunology and Department of Laboratory Medicine, Affiliated Hospital of Jiangsu University, 438 Jiefang Road, Zhenjiang 212001, China; ^2^Department of Pathogenic Biology, School of Medical Science and Laboratory Medicine, Jiangsu University, Zhenjiang 212013, China; ^3^Department of Immunology, School of Medical Science and Laboratory Medicine, Jiangsu University, Zhenjiang 212013, China

## Abstract

It has been reported that Notch family proteins are expressed in synovium tissue and involved in the proliferation of synoviocyte from rheumatoid arthritis (RA). The aim of this paper was to investigate whether Notch signaling mediated TNF-**α**-induced cytokine production of cultured fibroblast-like synoviocytes (FLSs) from RA. Exposure of RA FLSs to TNF-**α** (10 ng/ml) led to increase of Hes-1, a target gene of Notch signaling, and a marked upregulation of Notch 2, Delta-like 1, and Delta-like 3 mRNA levels. Blockage of Notch signaling by a **γ**-secretase inhibitor (DAPT) inhibited IL-6 secretion of RA FLSs in response to TNF-**α** while treatment with recombinant fusion protein of Notch ligand Delta-like 1 promoted such response. TNF-**α** stimulation also induced IL-6 secretion in OA FLSs; however, the Hes-1 level remained unaffected. Our data confirm the functional involvement of Notch pathway in the pathophysiology of RA FLSs which may provide a new target for RA therapy.

## 1. Introduction

Rheumatoid arthritis (RA) is characterized by chronic and progressive inflammation of multiple joints, resulting in leukocyte invasion, formation of pannus, progressive degradation of the cartilage, and erosion of the bones [1]. Although the exact mechanism of RA pathogenesis is not well defined, it has been suggested that activated synoviocytes may play an important role, primarily through proliferation in inflamed synovia, and production of proinflammatory cytokines, matrix metalloproteinases (MMPs), and chemokines [2]. Cytokines secreted by RA synoviocytes include tumor necrosis factor-*α* (TNF-*α*), interleukin-1*α* (IL-1*α*), interleukin-1*β* (IL-1*β*), interleukin-6 (IL-6), interleukin-8 (IL-8), and granulocyte macrophage-colony stimulating factor (GM-CSF) [3]. Among these cytokines, TNF-*α* appears to be the major proinflammatory cytokine because it is known to strongly induce the production of IL-6, IL-8, GM-CSF, and even itself in synoviocytes; however, the precise molecular mechanism of cytokines production in response to TNF-*α* stimulation is not clarified [4].

The Notch signaling pathway is highly conserved beyond species and plays a critical role in a variety of cellular functions, including cell proliferation, differentiation, and apoptosis [5]. To date, four Notch receptors (Notch 1–4) and five of their ligands (Delta-like 1, 3, 4; Jagged-1, 2) have been identified in mammals. Upon ligand binding, the intracellular domain (ICD) of the receptor is proteolytically cleaved and translocated into the nucleus, where it associates with the RBP-J*κ* transcription factor and regulates expression of several target genes, such as the basic helix-loop-helix (bHLH) proteins hairy-enhancer of split-like 1- (Hes-1) and Hes-5 [6].

Several early reports have shown the functional involvement of Notch pathway in the pathophysiology of RA synovitis. Ishii et al. and Yabe et al. showed that the expression pattern of Notch homologues among synovium from OA and RA patients differed from that of normal subjects [7, 8]. Another report demonstrated the expression of Notch-1 in synoviocytes and the presence of Notch-1 fragment in the nuclei of RA synoviocytes and suggested the involvement of Notch-1 signaling in the TNF-*α*-induced proliferation of RA synoviocytes [9]. TNF-*α* induced the expression of IL-6, MMP11, Notch-1, Notch-4, and Jagged-2 in RA FLSs [10, 11]. As yet, however, the expression pattern of Notch molecules on cultured synoviocyte is controversial and the relationship between increased expression of IL-6 or MMP11 and Notch signaling molecules response to TNF-*α*-stimulation remains unclear. In this study, we demonstrate that Notch signaling mediates TNF-*α*-induced secretion of IL-6 in RA FLSs while the expression pattern of Notch receptors and ligands upon TNF-*α*-stimulation differs from the previously reported.

## 2. Materials and Methods

### 2.1. Patients

Synovial tissue samples were obtained from patients with RA (*n* = 4, one male, three females, the mean age 54.7 ± 20.1) and OA (*n* = 3, one male, two females, the mean age 51.3 ± 10.4) at the time of total knee joint replacement. All RA patients fulfilled the respective American Rheumatism Association criteria [12]. All OA patients were evaluated by a certified rheumatologist and diagnosed based on the criteria developed by the ACR Diagnostic Subcommittee for OA [13]. Written consent was obtained from all patients after a full explanation of the procedure in conformity with requirements of the Committee on Ethics of Biomedicine Research of Affiliated Hospital of Jiangsu University.

### 2.2. Preparation of Fibroblast-Like Synoviocytes

RA FLSs were isolated from synovial tissues according to the method previously described [14]. Briefly, the collected synovial tissues were minced and cultured as explant pieces in a flask. Within 14 days, fibroblast-like cells migrated out from the tissue explants and formed confluent monolayers. The cells were collected by trypsinization and reseeded into flasks for expansion at 37°C in Dulbecco's modified Eagle medium (Gibco, BRL, USA) supplemented with 10% heat inactivated fetal calf serum (Gibco, BRL, USA), 100 units/mL penicillin, and 100 *μ*g/mL streptomycin. Fibroblasts between passages 3 and 5 were used for each experiment after being identified by morphology and purity analysis. Osteoarthritis (OA) FLSs were similarly prepared.

### 2.3. Treatment of FLSs with TNF-*α* and Notch Inhibitor and Ligand Fusion Protein

For kinetic analysis of the TNF-*α*-induced expression of Notch target gene Hes-1 and cytokines production, 1 × 10^5^ FLSs per well were seeded into 12-well culture plates and subsequently stimulated with TNF-*α* (10 ng/mL, R&D Systems, Abingdon, UK) for various times. After stimulation, culture supernatants were collected and kept at −80°C for the measurement of IL-6 and IL-8 by ELISA, while the cells were lysed in TRIzol reagent (Invitrogen, Carlsbad, CA, USA) for RNA isolation. To observe the effect of Notch signaling in the TNF-*α*-induced cytokine production, a Notch inhibitor, DAPT (Sigma, St. Louis, MO, USA) and Notch ligand fusion protein, Delta-like 1 (R&D Systems, Abingdon, UK), were also added into the culture system together with TNF-*α*.

### 2.4. Measurement of IL-6 and IL-8 by ELISA

The concentrations of IL-6 and IL-8 in culture supernatants were determined by using commercial ELISA kits (eBioscience, San Diego, USA) according to manufacturer's recommendations.

### 2.5. RNA Isolation and Real-Time RT-PCR

For the determination of mRNA expression of Notch receptors, ligands and target gene Hes1, total RNA was extracted from FLSs with or without stimulation by using TRIzol reagent (Invitrogen, Carlsbad, CA, USA). cDNA was prepared by reverse transcription with oligo (dT) from total RNA extraction. Real-time PCR for Notch signaling molecules and a reference gene (*β*-actin) was performed in a LightCycler instrument (Roche Molecular Diagnostics, Mannheim, Germany) with the SYBRgreen mastermix kit (Takara, Shiga, Japan). The target gene expression was then normalized relative to *β*-actin. Primers used were forward (5′-TCAGCGGGATCCACTGTGAG-3′) and reverse (5′-ACACAGGCAGGTGAACGAGTTG-3′) for Notch 1; forward (5′-TGCCAAGCTCAGTGGTGTTGTA-3′) and reverse (5′-TGCTAGGCTTTGTGGGATTCAG-3′) for Notch 2; forward (5′-GGTTCCCAGTGAGCACCCTTAC-3′) and reverse (5′-GTGGATTCGGACCAGTCTGAGAG-3′) for Notch 3; forward (5′-ACCTGCTCAACGGCTTCCA-3′) and reverse (5′-AGCTTCTGCACTCATCGATATCCTC-3′) for Notch 4; forward (5′-ACCTGCTCAACGGCTTCCA-3′) and reverse (5′-AGCTTCTGCACTCATCGATATCCTC-3′) for Jagged-1; forward (5′-ACCAGGTGGACGGCTTTGAG-3′) and reverse (5′-CCCGGGATGCAATCACAGTAATA-3′) for Jagged 2; forward (5′-TGGGCTACTCCGGCTTCAAC-3′) and reverse (5′-ACAGGTAGGCATCACCGAGGTC-3′) for Delta-like 1; forward (5′-TCAACAACCTAAGGACGCAGGAG-3′) and reverse (5′-CTACATCTTCAGGGCGATTCCAA-3′) for Delta-like 3; forward (5′-GTCCAACTGTGGCAAACAGCA-3′) and reverse (5′-AGCATATCGCTGATATCCGACACTC-3′) for Delta-like 4; forward (5′-GACTGTGAAGCACCTCCG-3′) and reverse (5′-GTCATGGCGTTGATCTGG-3′) for Hes1; forward (5′-GAAGTCCCTCACCCTCCCAA-3′) and reverse (5′-GGCATGGACGCGACCA-3′) for *β*-actin.

### 2.6. Statistical Analysis

Two-tailed Student's *t*-test was used for determining significant differences (*P* ≤ 0.05) between groups.

## 3. Results

### 3.1. TNF-*α* Stimulation Induces the Activation of Notch Signaling in Fibroblast-Like Synoviocytes (FLS) from RA

**Figure 1:**
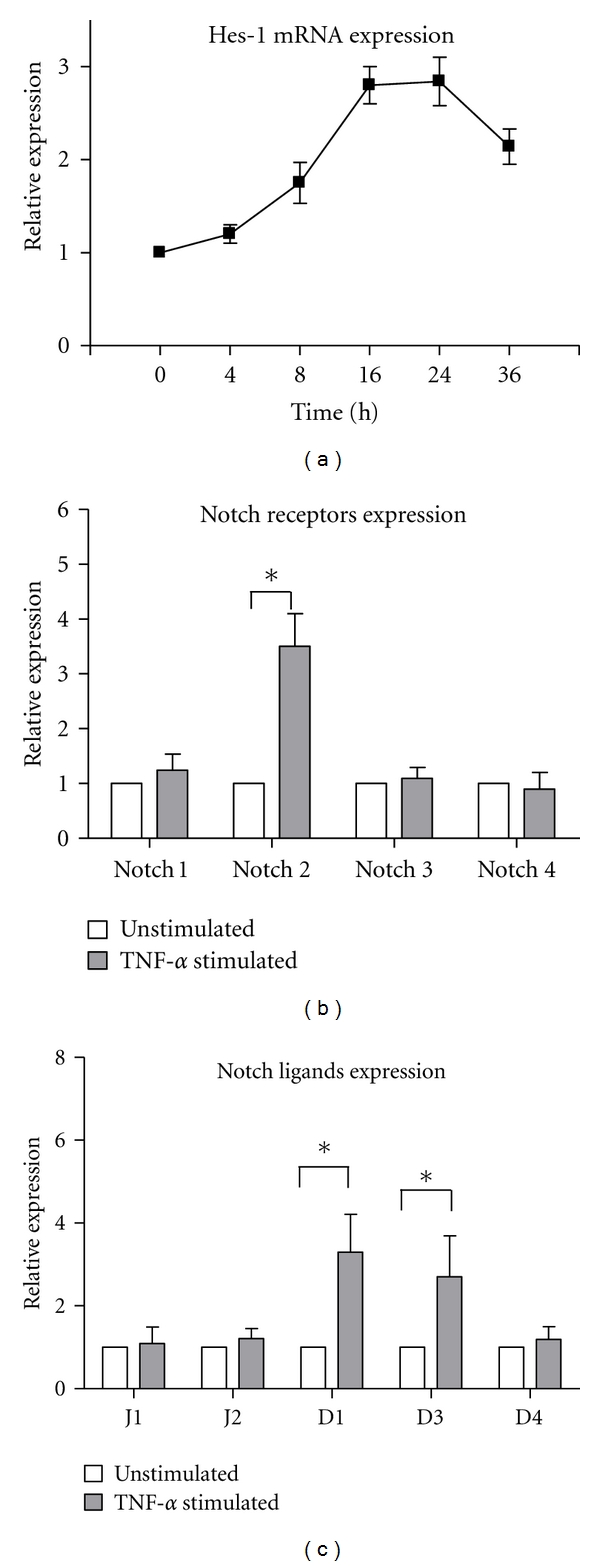
TNF-*α* stimulation induces the mRNA expression of Notch signaling molecules in fibroblast-like synoviocytes (FLS) from RA. (a) FLSs from 4 RA patients were cultured in the presence of TNF-*α* (10 ng/mL) for 0, 4, 8, 16, 24, or 36 h, and then analyzed for Hes-1 expression by PCR; (b) FLSs from 4 RA patients were cultured in the presence of TNF-*α* (10 ng/mL) for 24 h, followed by analysis of Notch receptors expression by PCR; (c) FLSs from 4 RA patients were cultured in the presence of TNF-*α* (10 ng/mL) for 24 h, and then analyzed for Notch ligands expression by PCR; J1: Jagged 1; J2: Jagged 2; D1: Delta-like 1; D3: Delta-like 3; D4: Delta-like 4. Results were normalized to unstimulated condition (basal value = 1). Each column is the mean ± SD. *P < 0.05 for unstimulated group versus TNF-*α*-stimulated group.

We first determined the effect of TNF-*α* on the expression of Notch signaling molecules in RA FLSs by real-time PCR. Exposure of RA FLSs to TNF-*α* led to a gradual, time-dependent increase of Hes-1 mRNA levels, a target gene of Notch signaling, which reached a maximum after 16–24 h of stimulation (Figure 1(a)). mRNAs of four receptors and five ligands of Notch signaling were also detected in RA FLSs stimulated with TNF-*α* (10 ng/mL) for 24 h. As shown in Figure 1(b), TNF-*α* stimulation induced a marked upregulation of Notch 2, in contrast, the levels of other three receptors were unchanged. TNF-*α* also significantly increased the mRNA expression of two ligands, Delta-like 1 and Delta-like 3, while the expression of the other three ligands was not affected (Figure 1(c)). These data suggest that Notch signaling is activated upon TNF-*α* stimulation in RA FLSs.

### 3.2. Notch Signaling Mediates TNF-*α*-Induced IL-6 Production in FLSs from RA

**Figure 2:**
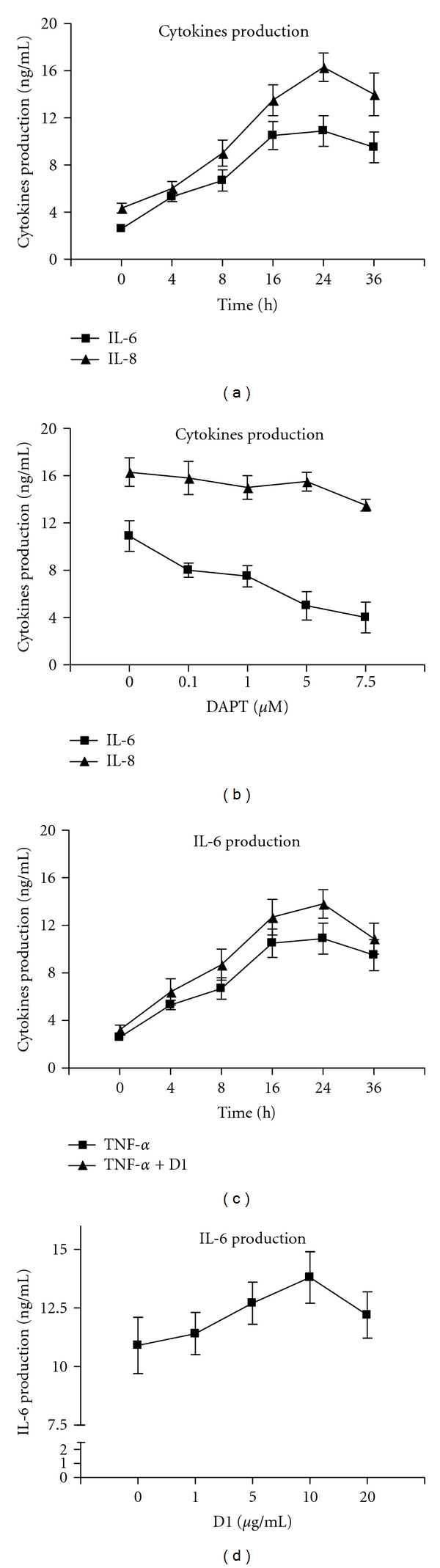
Notch activity contributes to TNF-*α*-induced IL-6 production of FLSs from RA. (a) FLSs from 4 RA patients were cultured in the presence of TNF-*α* (10 ng/mL) for 0, 4, 8, 16, 24, or 36 h, and then measured the levels of supernatant IL-6 and IL-8 by ELISA; (b) RA FLSs were cultured in the presence of TNF-*α* (10 ng/mL) in addition to the indicated concentration of DAPT (Notch signaling blocking reagent) for 24 h. Supernatant IL-6 and IL-8 were determined by ELISA; (c) RA FLSs were cultured in the presence of TNF-*α* (10 ng/mL) in addition to Delta-like 1 fusion protein (10 *μ*g/mL) for indicated times. Supernatant IL-6 was determined by ELISA; (d) RA FLSs were cultured in the presence of TNF-*α* (10 ng/mL) in addition to indicated concentrations of Delta-like 1 fusion protein for 24 h. Supernatant IL-6 was determined by ELISA. Values are the mean ± SD.

TNF-*α* stimulation induced a marked secretion of IL-6 and IL-8 from RA FLSs, which reached a maximum at 24 h of stimulation (Figure 2(a)). To test whether the activated Notch signaling mentioned above mediates this process, we first determined the effect of DAPT, a Notch signaling inhibitor, on TNF-*α*-stimulated cytokines secretion. Coincubation with such blocking reagent inhibited IL-6 secretion in response to TNF-*α* in a dose-dependent manner; however, DAPT did not significantly reduce the IL-8 secretion (Figure 2(b)). To confirm a role of Notch signaling as a mediator of TNF-*α*-induced IL-6 secretion, we examined the effect of human recombinant fusion protein of Notch ligand Delta-like 1 on TNF-*α*-induced IL-6 secretion. As demonstrated in Figure 2(c), when Delta-like 1 protein was added together with TNF-*α*, augmentation of IL-6 production was observed in a time-dependent manner. The facilitation of Delta-like 1 protein on IL-6 secretion was also dose-dependent with maximal response at 10 *μ*g/mL (Figure 2(d)).

### 3.3. Effect of TNF-*α* Stimulation on IL-6 and Hes-1 Production in OA FLSs

**Figure 3:**
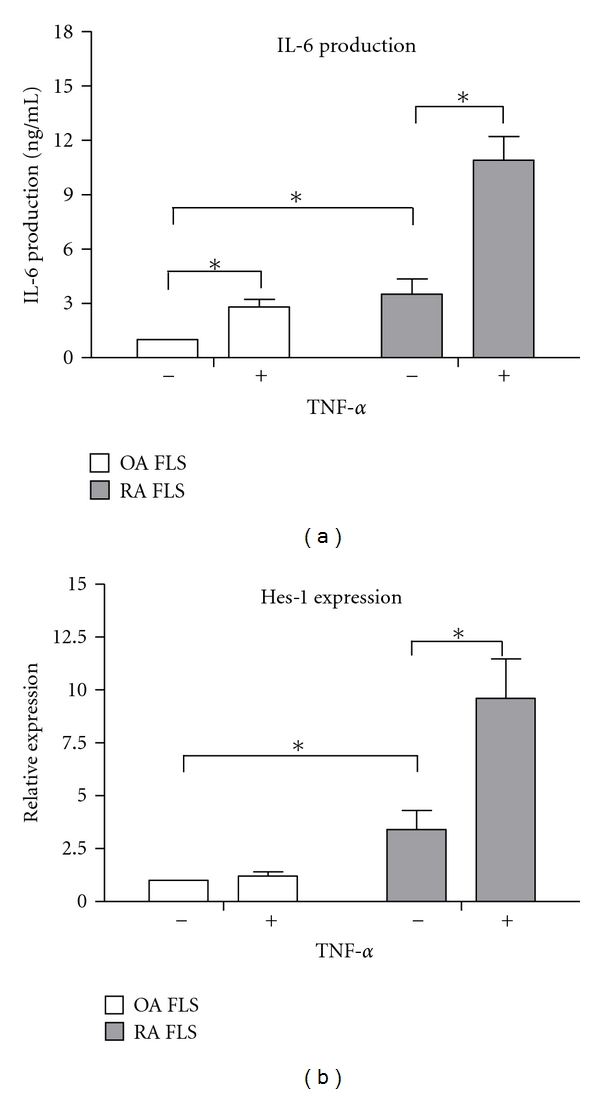
Effect of TNF-*α* stimulation on IL-6 and Hes-1 production in OA FLSs. (a) OA and RA FLSs were incubated with TNF-*α* (10 ng/mL) for 24 h, and then the supernatants were collected and analyzed for the IL-6 production by ELISA; (b) OA and RA FLSs were incubated with TNF-*α* (10 ng/mL) for 24 h, and then the cells were collected and analyzed for the Hes-1 mRNA expression by PCR. Each column is the mean ± SD. *P < 0.05.

To test whether Notch signaling could also mediate IL-6 production in OA FLSs, we determined the ability of TNF-*α* to induce IL-6 secretion and Hes-1 mRNA expression in primary human OA FLSs. The spontaneous secretion of IL-6 in cultured OA FLSs was significantly lower than that of RA FLSs, while TNF-*α* stimulation induced marked IL-6 secretion in both OA FLSs and RA FLSs (Figure 3(a)). The basal Hes-1 mRNA expression in OA FLSs was also significantly lower than that of RA FLSs; however, unlike in RA FLSs, TNF-*α* stimulation did not increase the Hes-1 mRNA expression in OA FLSs (Figure 3(b)). This result indicates that involvement of Notch signaling in TNF-*α*-induced IL-6 production should be unique to RA FLSs.

## 4. Discussion

Fibroblast-like synoviocytes, (FLSs) also called synovial fibroblasts (SF), are resident mesenchymal cells of synovial joints [15]. Activation of FLSs in the setting of RA leads to the production of a variety of cytokines, small molecule mediators of inflammation, and proteolytic enzymes which are responsible for the progressive destruction of articular cartilage and bone [4, 10]. Activation of FLSs could be initiated by cytokines, among which TNF-*α* is paramount. Activated FLSs by TNF-*α* in turn produce IL-6, IL-1*β*, and even itself to sustain regulatory feedback loops that perpetuate local joint inflammation. Such mechanism has been confirmed by the clinical efficacy of TNF-*α* blocking reagent in the treatment of RA synovitis. However, it remains less clear how many signal pathways are activated within FLSs upon TNF-*α* stimulation, and therefore which signal pathway may be the alternative target for clinical intervention.

Several early studies, which reported the expression of Notch molecules in RA synovium and the involvement of Notch signaling in the activation of cultured FLSs [9, 11, 16], led us to test the role of Notch signaling in the cytokine secretion of RA FLSs in response to TNF-*α* stimulation. The present study first observed that TNF-*α* stimulation led to a gradual, time-dependent increase of Hes-1 mRNA levels in RA FLSs, which reached a maximum after 16–24 h of stimulation. Hes-1 is the most well characterized, *γ*-secretase-dependent transcriptional target gene of Notch, and upregulated expression of Hes-1 represents the activated Notch signaling. A previous study has also demonstrated that TNF-*α* induced the elicitation of the Notch signaling by the observation of nuclear translocation of Notch intracellular domain (NICD) in cultured RA FLSs [9]. However, the same study reported that TNF-*α* treatment (200 pg/mL) upregulated the expression of Notch 1, Notch 4, and Jagged 2, which is quite different from our results that TNF-*α* stimulation (10 ng/mL) induced a marked upregulation of Notch 2 and two ligands, Delta-like 1 and Delta-like 3. This diversity may be due to the different stimulation concentration of TNF-*α* used in experimental system or the different culture times. Indeed, the expression profile of Notch receptors or ligands in RA local synovium tissue also varied among previous reports [7, 8, 11]. Nevertheless, here we confirmed the result that TNF-*α* could induce the activation of Notch signaling in cultured RA FLSs.

TNF-*α* has been shown to induce cytokine production in RA FLSs, such as IL-6 and IL-8 [3, 10, 11]. We also observed the increased production of IL-6 and IL-8 upon TNF-*α* stimulation, which was consistent with the previous reports. FLSs in the intimal lining have been shown to be the primary source of IL-6 by in situ hybridization and immunohistochemistry studies. Cultured RA FLSs spontaneously produce IL-6, and their production is markedly increased by TNF-*α*, thus, targeting the function of FLSs to produce IL-6 might improve clinical outcomes in inflammatory arthritis without suppressing systemic immunity [3, 10].

To test whether the activated Notch signaling mediate the cytokine production in response to TNF-*α* stimulation, DAPT, a *γ*-secretase inhibitor, was added into the culture system to block the activation of Notch signaling. We found that DAPT inhibited IL-6 secretion in response to TNF-*α* in a dose-dependent manner, however, it did not significantly reduce the IL-8 secretion. To confirm a role of Notch signaling as a mediator of TNF-*α*-induced IL-6 secretion, we also demonstrated that Delta-like 1 fusion protein added together with TNF-*α*, augmented IL-6 production in a time and dose-dependent manner. In our study, TNF-*α* stimulation could also induce marked IL-6 secretion in OA FLSs other than RA FLSs. However, unlike in RA FLSs, TNF-*α* stimulation did not increase the Hes-1 mRNA expression in OA FLSs. Such inconsistent result indicated that the targets of Notch-mediated transcriptional activation should be explored other than Hes-1. Alternatively, there may be target sequences of CSL in the IL-6 promoter. Similar research strategies had been reported in a newly published paper [17]. By ChIP analysis, Keerthivasan et al. report NICD directly binds to both ROR-*γ*t and IL-17 promoters and regulates Th17 differentiation (IL-17 induction). Further experimentation is required to test whether Notch can bind directly to the IL-6 promoters.

The function of FLSs to produce IL-6 has been reported to be regulated in an NF-*κ*B-dependent pathway. In fact, some effective anti-RA drugs are now known to inhibit NF-*κ*B and its activation cascade. Here we reported that Notch signaling also mediated TNF-*α*-induced IL-6 production in cultured RA FLSs which can also potentially serve as therapeutic target signaling. We have also demonstrated the activation of Notch signaling in helper T cells from RA patients [18]. Based on these considerations, using inhibitors of *γ*-secretase (as is already in use for the treatment of Alzheimer's disease) to block the activated Notch signaling might be a feasible approach to RA therapy [19].
